# The society for equity neuroscience (SEQUINS): Seeking to eliminate global brain health inequities and disparities through research

**DOI:** 10.1016/j.neuros.2025.100001

**Published:** 2025-04-22

**Authors:** Bruce Ovbiagele

**Affiliations:** Department of Neurology, University of California, 400 Parnassus, San Francisco, CA 94143, United States

**Keywords:** Brain health, Neurological disease, Health equity, Disparities, Epigenetics, Social determinants, Global

## Abstract

Over 3 billion people are affected by a neurological condition, which is now the leading cause of illness and disability worldwide. However, burden of neurological disease is unevenly distributed, with certain groups and regions being more affected than others. Neurological disorders disproportionately affect certain racial and ethnic communities (e.g., Black, Indigenous, and people of color), those in lower socioeconomic groups, individuals living in underserved geographies including rural areas and low-income countries, and other marginalized populations. These populations tend to have poorer neurological outcomes because they are adversely impacted by the social drivers of brain health including lack of access to care, minimal participation in pivotal neurological trials, and underrepresentation in neuroscience research careers. Equity neuroscience is a multidisciplinary academic field devoted to understanding inequities in the manifestations of nervous system health, from particles to people, at multiple interacting levels, with a bidirectional local to global scope. The Society for Equity Neuroscience (SEQUINS) founded in 2024 aims to enhance brain health around the world by improving equitable nervous system care and outcomes through research. SEQUIINS seeks to be the central and unifying global organization for equity neuroscientists; supporting, teaching, promoting, and applying high quality research to achieve equitable nervous system care and health. This paper highlights the rationale for establishing a dedicated community of equity neuroscientists and presents the mission, vision, and emerging academic activities of SEQUINS.

## Introduction

There are prominent differences in the ability of certain populations to achieve optimal neurological outcomes, and for several of these groups, these disparities continue largely unresolved and unexplained [1,2] Over the decades, while these disparities have been closely surveilled, repeatedly highlighted, with frequent calls to address them, not much has changed [3] What has prevented us from bending the neurological disease disparities curve for key demographic groups, especially the disproportionately higher neurologic mortality rates racial/ethnic minorities in high income countries and low/middle income countries? Moreover, groups overly affected by poor neurological outcomes are underrepresented in our medical and scientific workforces [4–6] Over the years, a variety of initiatives have been implemented to increase the numbers of historically underrepresented individuals in neurology, but the needle has barely moved [7] Why have these efforts to widen representation not gained any real traction? To be successful, we may need to operate with a specific rubric and within a dedicated community that fosters greater clarity, communication, consistency, consensus, relevance, and rigor.

Globally, gaps in neurological health and access to healthcare for neurologic disorders are enormous [8] However, a key distinction is frequently made between health inequities (i.e., differences in barriers which hamper individuals from achieving their greatest health potential) and health disparities (i.e., differences in processes and outcomes pertaining to in healthcare). Based on compelling evidence of health disparities and their adverse effect on health outcomes, the paradigm has shifted to emphasize translating knowledge about health disparities into solutions to attain health equity [9] A robust body of evidence points to individual level, environmental, and societal barriers to achieving brain health equity, but the evidence base for sustainable solutions is lacking [10,11] To move towards a world with sustained neurologic health equity will require a dedicated community of scientific professionals committed to conducting high quality neurologic health equity research, imparting training in health equity research methods, and translating efficacious interventions into effective solutions in the real world. ^4–6^

Recently, a proposal for a new discipline was made. Equity Neuroscience *“is the study of how the brain is mechanistically affected by health inequities, as well as the distinctive barriers and facilitators to optimal functioning of the nervous system”* for everyone, every time, and everywhere [12] Equity Neuroscientists are envisioned to traverse all branches of science, stages of research, forms of neurological disease, and types of professional discipline. Presently, no organization has as its primary mission to bring together (and sustain) diverse collaborators across all scientific communities, sectors, disciplines, career stages, and neurological disease entities, to foster synergies, generate innovative solutions, and guide policy actions, which ultimately will drive equity in brain care and brain health, globally [12] To develop and promote this discipline, a new professional organization called the Society for Equity Neuroscience (SEQUINS) was established. This paper shares with the global neurological community, the emerging strategic directions, and principal activities of SEQUINS.

## Society for equity neuroscience

### 1. Statements, Membership, and Leadership

SEQUINS aims to nurture and grow an intellectual, interconnected, and international community, fully engaged in bold, new, and synergistic activities that move the field forward; and to translate discoveries and solutions into better brain health and longer lives in the real world for disparate populations. Table 1 displays the mission, vision, values, and goals of the organization. The emphasis is on establishing a sustainable scientific coalition involving stakeholders in academia, governments, foundations, industry, and the lay public. SEQUINS is a United States registered nonprofit (public charity), welcomes increased research expenditures to improve brain health outcomes [13], and its membership is open to persons from around the world who have demonstrated a commitment to promoting Equity Neuroscience research, education, and application. The Society was launched in June 2024 and currently has 77 members and rising. The inaugural board of directors is led by a multidisciplinary, multisectoral, multinational, and multicultural group of seasoned scientists and leaders (Table 2) [14]

### 2. Annual Meeting

The annual SEQUINS scientific meeting, is envisioned to be a multidisciplinary one-day scientific forum, held in Spring each year, focused on nervous system health and care, with the overarching goal of reducing neurological inequities and disparities, and accelerating the translation of research findings to improve outcomes for disproportionately burdened populations. The forum brings together leaders in the field of brain health equity research to review current “state-of-the-art” scientific work and formulate new directions in research and therapies. The unique composition of meeting allows for cross-fertilization between scientists, both young and established. It capitalizes on the interdisciplinary experience of the invited participant body and seeks to elucidate the evolution of a few major themes integral to the field. The topics for the individual talks and debates are chosen for their strategic impact on furthering understanding of underlying mechanisms and the development of innovative approaches for advancing brain health equity (Table 3). Each SEQUINS annual meeting will emphasize 5 major types of inequities/disparities (race/ethnic, sex/gender, geographic, socioeconomic, global).

SEQUINS annual meetings will identify knowledge gaps and controversies as well as present opportunities and solutions for researchers from diverse scientific and professional disciplines to discuss cutting-edge research about key inequities/disparities in brain care and health. Invited presentations will cover various aspects of investigation and management including basic/translational science, clinical science, population health, community engagement, clinical trials, implementation science, and policymaking/scaling-up. Representatives from academia, industry, the National Institutes of Health will participate in the robust discussions. Invited speakers will be required to present new, late breaking, and often unpublished data to stimulate energetic discussion, and the sessions will be designed to allow for substantial time for moderated general participant discussion. Networking functions will be embedded into scheduled events at the Meeting. The proceedings of the conference will be summarized and published in a scientific journal. Publishing the proceedings in a scientific journal augments the power of the meeting through peer review and dissemination to all a journal’s readers, rather than just the meeting attendees.

Effectiveness of each yearly SEQUINS meeting will be assessed by monitoring number of attendees and attendee engagement feedback. The annual SEQUINS scientific meeting is a collaborative initiative between the Society for Equity Neuroscience, and the Department of Neurology and the College of Graduate Studies at the Medical University of South Carolina (MUSC), as well as other stakeholders and partners as identified. The inaugural SEQUINS scientific meeting will be held on May 15th, 2025, in Charleston, South Carolina. An Annual Meeting Program Committee will provide the forum for conference development, management, and evolution. Each member of the program committee was selected based on expertise in key and complementary areas of equity neuroscience research. This will be the think-tank and workhorse committee guiding all aspects of implementation and evaluation of the SEQUINS annual meeting. The tentative agenda for SEQUINS 2025 can be seen in Table 4. Starting with the SEQUINS 2026 annual meeting, the program agenda will be tailored to incorporate themes and speakers that cover pivotal strategic plan health equity Recommendations (https://www.neurology.org/toc/wnl/101/7_Supplement_1) and will include a poster session for very early career individuals interested in equity neuroscience.

### 3. Scientific Journal

SEQUINS launched this journal, Equity Neuroscience, in 2025. With the motto “Impact through Insight”, the journal seeks to be the leading peer-reviewed open access online journal, that publishes the scholarly progress of work to understand, address, and ultimately eliminate neurological inequities based on sex, race, ethnicity, geography, and socioeconomic status. Equity Neuroscience intends to provide authoritative interdisciplinary and interprofessional information about neurological inequities among underserved and vulnerable populations with the goal of providing optimal outcomes and ultimately brain health equity for all. Its coverage will range from basic science to translational research to prevention, diagnosis, treatment, and management of neurological disease. The Journal will also serve as a primary resource for organizations, policymakers, and individuals who serve neurologically disparate populations at the community, state, regional, tribal, national, and international levels. A key emphasis is the publication of high-quality, high-impact, and innovative research articles in all areas related to brain health disparities

In Equity Neuroscience, efforts to explore underlying causes of neurological inequities and to describe interventions that have been undertaken to address major neurological inequities will be featured along with promising studies that serve as lessons for best practices in eliminating neurological inequities. The journal will cover all aspects of neurological inequities including social, cultural, behavioral, environmental, genetic, and epigenetic determinants contributing to differences in neurological disorder incidence, prevalence, death, survivorship, and burden of neurological diseases that exist among different populations around the world. EQN will prioritize articles that promote multi-, inter-, and trans-disciplinary research and foster a scholarly community with different expertise and resources, to develop new, synergistic perspectives. It is expected that the audience for EQN will include neurologists, psychiatrists, neurosurgeons, internists, pediatricians, geriatricians, basic/translational neuroscientists, epidemiologists, population health scientists, health services researchers, health equity researchers, global health researchers, and health policy researchers.

### 4. Other Programs and Initiatives

SEQUINS has developed several high-quality, evidence-based educational opportunities that are designed to advance learner competence in treatment of diseases of the nervous system, and to provide the latest neuroscientific information on brain health equity in a forum which encourages critical thinking and debate and fosters creative thinking for further research and academic pursuit. Its continuing education activities include conferences, symposia, seminars, debates, clinical presentations, research presentations, and other possible forums. The educational design, instructional method and learning format for each event is selected to best serve the measurable educational needs and learning objectives of the planned educational activity [15] The activities presentation methodology may include a one person delivered lecture with power point slides, interactive demonstrations, panel discussions, poster sessions, or breakout groups. Table 5 shows a select listing, description and rationale for various initiatives being developed or planned over the next two years under the auspices of SEQUINS.

## Conclusion

Neurological diseases have now overtaken cardiovascular diseases to be the world’s leading cause of morbidity and morbidity, primarily due to increasingly disparate outcomes across the globe [16] Many of these inequities and disparities in global brain health, are not necessarily new, appear to disproportionately affect certain demographic groups, and are projected to worsen with time [17] As such the time is now to develop new strategies to bolster the science and scientists required to fully understand the drivers and consequences of these inequities and disparities and identify evidence-based solutions for scaling and sharing [17–19] SEQUINS was borne out of a desire to do something different, i.e., foster a distinct, collegial, and interactive worldwide community of diverse researchers and their partners, collectively focused on bringing a sense of urgency, initiative, collaboration, and thoroughness to *“advance brain health equity through science”*.

## Figures and Tables

**Table 1:** Society for Equity Neuroscience Statements.

Statement Description	

**Mission**	• To advance brain health equity through science
**Vision**	• To be the central and unifying global organization for equity neuroscientists
**Values**	• Integrity • Innovation • Inclusion • Inspiration • Impact
**Tagline**	• *“Because All Brains Matter”*
**Goals**	• Support the equity neuroscience community and advance scientific exchange within it • Establish partnerships with key stakeholder organizations and groups • Identify and address key questions that would greatly advance brain health equity • Provide programs that enhance knowledge and inspire investigators • Ensure a diverse community of equity neuroscientists • Engage the public and advocate for equity neuroscience • Develop funding sources for working capital, staff support, and key programs

**Table 2:** Society for Equity Neuroscience Board.

Name	Role	Affiliation

Bruce Ovbiagele, MD	President	University of California, San Francisco
Lilyana Amezcua, MD	Vice President	University of Southern California
Kenneth Maynard, PhD	Treasurer	Takeda Pharmaceuticals
Karen Orjuela, MD MS	Secretary	University of Colorado
Gabriele C. De Luca, MD	Director	University of Oxford
Menghis Bairu, MD	Director	Bio Usawa Biotechnology Research
Mai Nguyen-Huynh, MD	Director	Kaiser Northern California
Anjail Sharrief, MD	Director	University of Texas, Houston
Na Tosha Gatson, MD PhD	Director	Indiana University
Janet Prvu Bettger, PhD	Director	Duke University
Carolina Maciel, MD MS	Director	University of Florida
Chiadi Onyike, MD	Director	Johns Hopkins
Meeryo Choe, MD	Director	University of California, Los Angeles

**Table 3:** Society for Equity Neuroscience Annual Meeting Topline Events.

Event	Description	Criteria/Miscellaneous

**Hall of Fame**	• Nominated by a Member of SEQUINS • Recognizes transformative career contributions to equity neuroscience research • Induction during SEQUINS Annual Meeting • Each Inductee will give an overview of their work, discuss prevailing gaps/opportunities, and vision for their field • No more than five inductees each year • Inductees will be memorialized in virtual hall of fame on the SEQUINS website	• Member of SEQUINS at the time of nomination • Selection by SEQUINS program committee • Candidate must be a senior investigator of any scientific (basic, translational, clinical, or population) or professional background who has made outstanding contributions in the field of equity neuroscience in their lifetime • Evidence of mentorship of students, post-docs, residents, fellows, and early career faculty is important • Presentations and induction must be done in person.
Outstanding Mentorship Award	• Recognizes excellent achievements in the mentoring of future generations of researchers in the field of equity neuroscience. • Although recipients are expected to be successful scientists, the award recognizes mentorship rather than scientific accomplishment, emphasizing the training experiences and successes of the nominee’s mentees, not the mentor’s personal career achievements. • The award acknowledges the successful development of mentees who will also go on to become independent equity neuroscientists, ultimately shaping the future of the field. • For this award, mentoring refers to the process of guiding, supporting, and promoting the training and career development of others. • Award recipients will be individuals who have actively engaged in sustained efforts aimed at the mentoring of trainees in the field of equity neuroscience. • Award is given during the annual SEQUINS meeting • Awardee will give a presentation	• Member of SEQUINS at the time of nomination • Selection by SEQUINS program committee • Nominees may include basic, clinical, or translational researchers working in the field of equity neuroscience. They should have a record of successfully mentoring researchers in the field of equity neuroscience over time. • Mentees should be actively involved in research, teaching, mentoring, community activities or other leadership activities in the field of equity neuroscience. • Nominated by a Member of SEQUINS who is/are mentee(s) of the nominee who have personal knowledge & first-hand experience of the nominee’s mentoring efforts. • Previous recipients of this award are ineligible.
Early Career Showcase Lecture	• Invited lecture by early career individual who participated in a funded training program and who is currently funded to study one of the five major areas of neurological inequities/disparities (sex/gender, race/ethnic, geographic, socioeconomic, or global) • Examples include graduates of funded training programs	• Member of SEQUINS at the time of nomination • Selection by SEQUINS program committee • Previous invitees are ineligible.
TRANSCRENDS Graduates	• Individuals graduating from the program will be named and recognized highlighting their affiliations, area(s) of research interest, and accomplishments	• Introductions by member of program leadership
TALENTS Graduates	• Individuals graduating from the program will be named and recognized highlighting their affiliations, area(s) of research interest, and accomplishments	• Introductions by member of program leadership
*Moving on Up: Succeeding as an Early Career Researcher*	• Early Career scientists in the audience In-person and online) will be invited to ask individual queries about building momentum in an academic career especially one tailored to pursuing equity neuroscience research.	• Moderated by a program committee member • Questions addressed by a panel of 2–3 senior equity neuroscience researchers who have a successful track record of high-quality research funding
*Speed Dating: Finding the Right Mentor & Collaborator*	• Early Career scientists in the audience In-person and online) will be invited to ask individual queries about building momentum in an academic career especially one tailored to pursuing equity neuroscience research.	• Moderated by a program committee member • Questions addressed by a panel of 2–3 senior equity neuroscience researchers who have served (or is serving) as a department chair or associate dean
Agency Briefings	• Brief presentation by a Program Officer from a key governmental or non-governmental research or public health agency	• Program Officer will present agency’s plans for equity neuroscience training, career development, and research as well as its contemporary funding opportunities

**Table 4:** Society for Equity Neuroscience Inaugural Annual Meeting Agenda.

Time	Topic	Speaker/Participants

8:30 am	**Networking Breakfast**	ALL
9:25 am	**Welcome Remarks**	Chair
9:30 am	*Hall of Fame Presentation I*	Robert Adams, MD
9:50 am	Q & A	ALL
9:55 am	*Hall of Fame Presentation II*	Moria Kapral, MD
10:15 am	Q & A	ALL
10:20 am	*Bright Star Award Lecture*	Ece Bayram MD
10:40 am	Q & A	ALL
10:45 am	*Succeeding as an Early Career Researcher*	Alexis Simpkins, MD Federico Rodriguez-Porcel, MD
11:05 am	**Refreshments Break**	ALL
11:20 am	*Recognitions of TRANSCENDS Graduates*	Program Leaders
11:35 am	*Hall of Fame Presentation III*	Cheryl Bushnell, MD
11:55 am	Q & A	ALL
12:00 pm	**Networking Lunch**	ALL
1:00 pm	*Hall of Fame Presentation IV*	Lewis Morgenstern, MD
1:20 pm	Q & A	ALL
1:25 pm	*Patrick A. Griffith Outstanding Mentor Presentation*	Deanna Saylor, MD
1:45 pm	Q & A	ALL
1:50 pm	**Refreshments Break**	ALL
2:05 pm	*Hall of Fame Presentation V*	Barbara Vickrey, MD
2:25 pm	Q & A	ALL
2:30 pm	*Funding Agency Briefing*	Richard Benson, MD
2:50 pm	Q & A	ALL
2:55 pm	*Finding the Right Mentor & Collaborator*	Daniel Lackland, DrPh Souvik Sen, MD Mark Stacy, MD
3:15 pm	*Recognitions of TALENTS Graduates*	Program Leaders
3:30 pm	*Induction of2025 Hall of Famers*	SEQUINS Leaders
4:00 pm	**Closing Remarks**	Co-Chair
4:30 pm	**Networking Social Reception**	ALL

**Table 5:** Society for Equity Neuroscience Select Programs and Initiatives

Initiative	Description	Rationale/Approach

***Bimonthly State of Science (SOS) Webinars***	• To highlight recent advancements in equity neuroscience research • Scientific lectures by eminent equity neuroscientists • One-hour Zoom-based Session	***Issue:*** • Insufficient avenues to promote recent and ongoing equity neuroscience research • Since many researchers are siloed within their phase of research (basic, translational, clinical, population) or neurological specialty, an opportunity to systematically expose the community to what is going on in various areas ***Method:*** • Create a systematic and rotational roster covering key areas sequentially ***Access:*** • SOS Webinars will be open to members only
***Annual INSPIRE Webinar***	• To highlight the pivotal research work of the underrepresented in science individuals to the broader	***Invited Presenter Eligibility:*** • Membership in SEQUINS
**I**ntroduction to **N**euroscientists **S**ucceeding and **P**rogressing with **I**nnovative **R**esearch **E**ndeavors	neuroscience and neurology community • To inspire URiMs who are early in their careers to conduct high-quality neurological research • To encourage URiM early career neuroscientists through the example of established URiM scientists	• Seven years or more from first academic faculty appointment • Current or Previous Independent Principal Investigator of Major Grant focused on an area of Neuroscience • Google H-Index ≥ 30 • Peer Review Publications ≥ 30 • Identification with a historically underrepresented in medicine group (i.e., related to race, ethnicity Black or African American, Hispanic, or Latino, American Indian or Alaska Native, Native Hawaiian and other Pacific Islanders)....OR... • Primarily based (i.e. reside more than 6 months out of a given year) in a low to middle income economy country as defined by the World Bank (please see https://datahelpdesk.worldbank.org/knowledgebase/articles/906519-world-bank-country-and-lending-groups) ***Invited Presenter Identification:*** • Self-Nominations • Nominations by Others • Invitations by Program Committee ***Program Agenda:*** • Virtual platform • Could be all lectures in one or half-day/sitting OR one/two one-hour/45 minute lecture(s) weekly over a given month each year ***Four to six invited presenters*** • At least 25% women • At least 25% from LMICs • At least 25% basic/translational researcher • At least 25% mid-career • Talks will focus on both the work and r'ourney of the scientist ***Access:*** • INSPIRE Webinars will be open to members (live) and recordings posted on website for public
***Fellow Membership***	• Recognize accomplishments and contributions to SEQUINS via higher tier membership level	• Fellow of the Society of Equity Neuroscience • With Fellow membership, offering use of a postnominal credential (FSEqN)
***Mentoring Program***	• Leverage membership for longer term mentoring of very early career individuals especially those from underrepresented groups	• Identify, match, and assign mentors for promising high school and undergraduate students interested in future careers in science or medicine • Develop virtual and/or in-person options
***Scientific and Policy Statements***	• Lingering Gaps • Guidance needed	• To be published in Equity Neuroscience • Multidisciplinary Writing Panels
***Scholarships***	• Financial support for students	• Medical Students • Graduate students in epidemiology, public health, heath services research, psychology, and sociology
***Pilot research funds***	• Very early career individuals may benefit from research seed monies to jumpstart their careers	• Seek Foundation (RWJ, Commonwealth fund, etc) and industry monies to support
***Community programs***	• Disparate communities need more information, outreach, and relationship building	• Develop signature models for implementing collaborations between local scientists and key stakeholders in disparate communities • **Community Health Advocate Training in Science (CHATS) Program** • **Brain Health and Wellness Science Fair** • Minoritized Patient Brain Science Education Materials
***Pan African Neurological Alliance for Clinical and Experimental Activities (PANACEA)***	• Under the auspices of SEQUINS, the PANACEA Network aims to conduct studies of treatments for neurological diseases in Africa through partnerships with governments, academia, private foundations, and industry. • PANACEA will establish a one-time contract for project participation, and use of a centralized Institutional Review Board.	• Network provides a robust, standardized, and accessible infrastructure to facilitate the rapid development and implementation of protocols in neurological disorders, in both adult and pediatric populations. • By bringing together expert investigators and experienced clinical sites across Africa, PANACEA is designed to efficiently conduct small and large clinical trials and research studies to advance neurological treatment, prevention, and recovery across the lifespan. • PANACEA will also collaborate with investigators and sites outside of Africa, and provide an educational platform for African neurological providers, neurological researchers, clinical trial coordinators and community stakeholders.
***No Brain Behind***	• Need to develop and reinforce a wider community of equity neuroscientists and their supporters using contemporary social media channels	• A podcast aimed at cultivating a consequential community of individuals interested in, inspired by, and informed about the science of brain health equity, through candid, collegial, and continuous conversations. • Dynamic discussions with article authors, funded investigators, award recipients, organization leaders, and policy makers, about the latest developments in and future directions of equity neuroscience, as well as glean career wisdom from these guests for our early career listeners.
***eNewsletter***	• Open and sustain regular communication channel with membership	• Monthly eNewsletter with news of research, news about members, and news with broader implications for equity neuroscience

## References

[1] Owolabi MO, Ovbiagele B. Global clinical neuroscience: journey of a thousand miles begins with a single step. J Neurol Sci 2014;344(1–2):223 Sep 15. 24972817 10.1016/j.jns.2014.06.011 PMC4143427

[2] Woolf SH, Chapman DA, Lee JH, Johnston KC, Benson RT, Trevathan E, Smith WR, Gaskin DJ. The Lives Lost to Inequities: Avertable Deaths From Neurologic Diseases in the Past Decade. Neurology 2023;101 7 Suppl 1):S9–S16. 37580146 10.1212/WNL.0000000000207561 PMC10605951

[3] Prust ML, Forman R, Ovbiagele B. Addressing disparities in the global epidemiology of stroke. Nat Rev Neurol 2024;20(4):207–21 Apr. 38228908 10.1038/s41582-023-00921-z

[4] Eyre HA, Hynes W, Ayadi R, Manes F, Swieboda P. Brain capital is crucial for global sustainable development. Lancet Neurol 2024;23(3):233–5 Mar. 38280391 10.1016/S1474-4422(24)00031-0

[5] Ovbiagele B, Amezcua L, Cruz-Flores SC, Griffith P, Jean-Louis G, Jenkins C, Howard VJ, Neurology Smith-Byrd G. Health Disparities Research Curricula and Training Development: Recommendations From a, 101. National Institute of Neurological Disorders and Stroke Workgroup; 2023. p. S47–58. 10.1212/WNL.0000000000207564 37580153

[6] Lizarraga KJ, Gyang T, Benson RT, Birbeck GL, Johnston KC, Royal W 3rd, Sacco RL, Segal B, Vickrey BG, Griggs RC, Holloway RG. Seven Strategies to Integrate Equity within Translational Research in Neurology. Ann Neurol 2024;95(3):432–41 Mar. 38270253 10.1002/ana.26873 PMC10922988

[7] Snyder JE, Upton RD, Hassett TC, Lee H, Nouri Z, Dill M. Black Representation in the Primary Care Physician Workforce and Its Association With Population Life Expectancy and Mortality Rates in the US. JAMA Netw Open 2023;6(4):e236687. 37058307 10.1001/jamanetworkopen.2023.6687 PMC10105312

[8] Winter SF, Walsh D, Catsman-Berrevoets C, Feigin V, Destrebecq F, Dickson SL, Leonardi M, Hoemberg V, Tassorelli C, Ferretti MT, Dé A, Chadha AS, Lynch C, Bakhtadze S, Saylor D, Hwang S, Rostasy K, Kluger BM, Wright C, Zee PC, Dodick DW, Jaarsma J, Owolabi MO, Zaletel J, Albreht T, Dhamija RK, Helme A, Laurson-Doube J, Amos A, Baingana FK, Baker GA, Sofia F, Galvin O, Hawrot T. National plans and awareness campaigns as priorities for achieving global brain health. Lancet Glob Health 2024;12(4):e697–706 Apr. 38485433 10.1016/S2214-109X(23)00598-3 PMC10951964

[9] Ovbiagele B HEADS-UP: Understanding and Problem-Solving: Seeking Hands-Down Solutions to Major Inequities in Stroke. Stroke 2020;51:3375–81. 33104464 10.1161/STROKEAHA.120.032442

[10] Sisodiya SM, Gulcebi MI, Fortunato F, Mills JD, Haynes E, Bramon E, Chadwick P, Ciccarelli O, David AS, De Meyer K, Fox NC, Davan Wetton J, Koltzenburg M, Kullmann DM, Kurian MA, Manji H, Maslin MA, Matharu M, Montgomery H, Romanello M, Werring DJ, Zhang L, Friston KJ, Hanna MG. Climate change and disorders of the nervous system. Lancet Neurol 2024;23(6):636–48 Jun. 38760101 10.1016/S1474-4422(24)00087-5

[11] Sarfo FS, Ovbiagele B. Utilizing Implementation Science to Bridge Cerebrovascular Health Disparities: a Local to Global Perspective. Curr Neurol Neurosci Rep 2022;22(5):293–303 May. 35381952 10.1007/s11910-022-01193-8 PMC9081275

[12] Ovbiagele B A proposal for equity neuroscience. Lancet Neurol 2024;23(9):862–3 Sep. 39152024 10.1016/S1474-4422(24)00317-X

[13] Kim YD, Jung YH, Norrving B, Ovbiagele B, Saposnik G. Does national expenditure on research and development influence stroke outcomes? Int J Stroke 2017;12(8):827–34 Oct. 28355959 10.1177/1747493017702667

[14] Esenwa C, Ovbiagele B. Paging Global Neurology Organizations-The Time Is Now for More Inclusive Leadership. JAMA Neurol 2022;79(10):964–5. 35939310 10.1001/jamaneurol.2022.2181

[15] Riddell MA, Edwards N, Thompson SR, Bernabe-Ortiz A, Praveen D, Johnson C, Kengne AP, Liu P, McCready T, Ng E, Nieuwlaat R, Ovbiagele B, Owolabi M, Peiris D, Thrift AG, Tobe S, Yusoff K. GACD Hypertension Research Programme. Developing consensus measures for global programs: lessons from the Global Alliance for Chronic Diseases Hypertension research program. Global Health 2017;13(1):17 Mar 15. 28298233 10.1186/s12992-017-0242-8 PMC5353794

[16] GBD 2021 Nervous System Disorders Collaborators Global, regional, and national burden of disorders affecting the nervous system, 1990–2021: a systematic analysis for the Global Burden of Disease Study 2021. Lancet Neurol. 2024;23(4):344–81. 38493795 10.1016/S1474-4422(24)00038-3 PMC10949203

[17] Winkler AS, Gupta S, Patel V, Bhebhe A, Fleury A, Aukrust CG, Dua T, Welte TM, Chakraborty S, Park KB. Global brain health-the time to act is now. Lancet Glob Health 2024;12(5):e735–6 May. 38493787 10.1016/S2214-109X(23)00602-2

[18] Rost NS, Salinas J, Jordan JT, Banwell B, Correa DJ, Said RR, Selwa LM, Song S, Evans DA. The Brain Health Imperative in the 21st Century-A Call to Action: the AAN Brain Health Platform and Position Statement. Neurology 2023;101(13):570–9. 37730439 10.1212/WNL.0000000000207739 PMC10558159

[19] Grisold W, Dodick DW, Guekht A, Lewis SL, Wijeratne T. World Brain Day 2024: a focus on brain health and prevention. Lancet Neurol 2024;23(9):863–4 Sep. 39033775 10.1016/S1474-4422(24)00270-9

